# Keratoacanthoma, committed stem cells and neoplastic aberrant infundibulogenesis integral to formulating a conceptual model for an infundibulocystic pathway to squamous cell carcinoma

**DOI:** 10.1111/cup.13861

**Published:** 2020-10-16

**Authors:** Steven Kossard

**Affiliations:** ^1^ Kossard Dermatopathologists Laverty Pathology Macquarie Park New South Wales Australia

**Keywords:** BRAF inhibitors, infundibulocystic, keratoacanthoma, PD‐1 inhibitors, squamous cell carcinoma, stem cells

## Abstract

Keratoacanthomas (KAs) are distinctive tumors that are defined by their clinical and histopathological features. Their relationship and distinction from squamous cell carcinoma (SCC), however, remain controversial. All cytogenic and immunohistochemical markers that have been applied in this quest have failed. A close relationship of KAs to hair follicles has been recognized. The descriptive term infundibulocystic or infundibular SCC was introduced to define a more broad‐based pathway encompassing KAs. The follicular infundibulum roles in respect to neoplasia and wound healing are important elements in understanding the pathogenesis of KAs. Mouse models for KA have provided insights into the relationship of KA to follicles and SCCs. These advances and together with the diverse clinical and histopathological aspects of KA have contributed to the formulation of a conceptual pathway. The central element is that ultraviolet (UV)‐mutated or activated committed infundibular stem cells are driven by the combination of a mutated oncogenic *RAS* pathway linked with the Wnt/beta‐catenin pathway responsible for stem cell maintenance, hair follicle development, wound healing and driving KA proliferation and terminal keratinization. The existence and activation of this mutated pathway may form the basis of the paradoxical emergence of KAs and SCCs in patients receiving BRAF and PD‐1 inhibitor therapy.

## 1 INTRODUCTION

Keratoacanthomas (KAs) are distinctive tumors that have been defined by their clinical and histopathological features, but their relationship to hair follicles and their distinction from squamous cell carcinoma (SCC) remain a controversy. The descriptive term infundibulocystic or infundibular SCC was introduced to define a more broad‐based pathway to SCC encompassing KAs and incorporate the follicular infundibulum as an essential element to the process.^1^

A broad range of biomarkers, particularly cytogenic markers and immunohistochemistry including CD123 have been unsuccessful in the quest to distinguish KA from SCC.^2^ This may reflect the fact that both KAs and SCCs are chronic UV‐induced squamoproliferative tumors sharing the same driver *RAS* mutations for neoplasia.^3^

Early carcinogen‐induced KA mouse models^4^ to show their relationship to follicles have been augmented by genetically engineered models.^5^ Significant advances have been made in defining skin stem cells that underpin both the homeostasis and dynamic physiology of follicles and the interfollicular epidermis.^6^, ^7^, ^8^ Advances in stem cell biology in humans have been reviewed in detail in respect to epidermal regeneration and carcinogenesis^9^ and have provided an opportunity to formulate a conceptual model within the framework of the infundibulocystic pathway to SCC. The central element is the concept of UV‐mutated or activated committed infundibular stem cells that are driven by the combination of two major molecular pathways, namely the mutated oncogenic *RAS* pathway linking KA and squamous cell carcinoma and the Wnt/beta‐catenin pathway responsible for skin stem cell maintenance, hair follicle development, wound healing and driving KA proliferation and terminal keratinization.^10^, ^11^

## 2 INFUNDIBULAR AND INFUNDIBULOCYSTIC ONCOGENIC PATHWAY TO SCC

**FIGURE 1:**
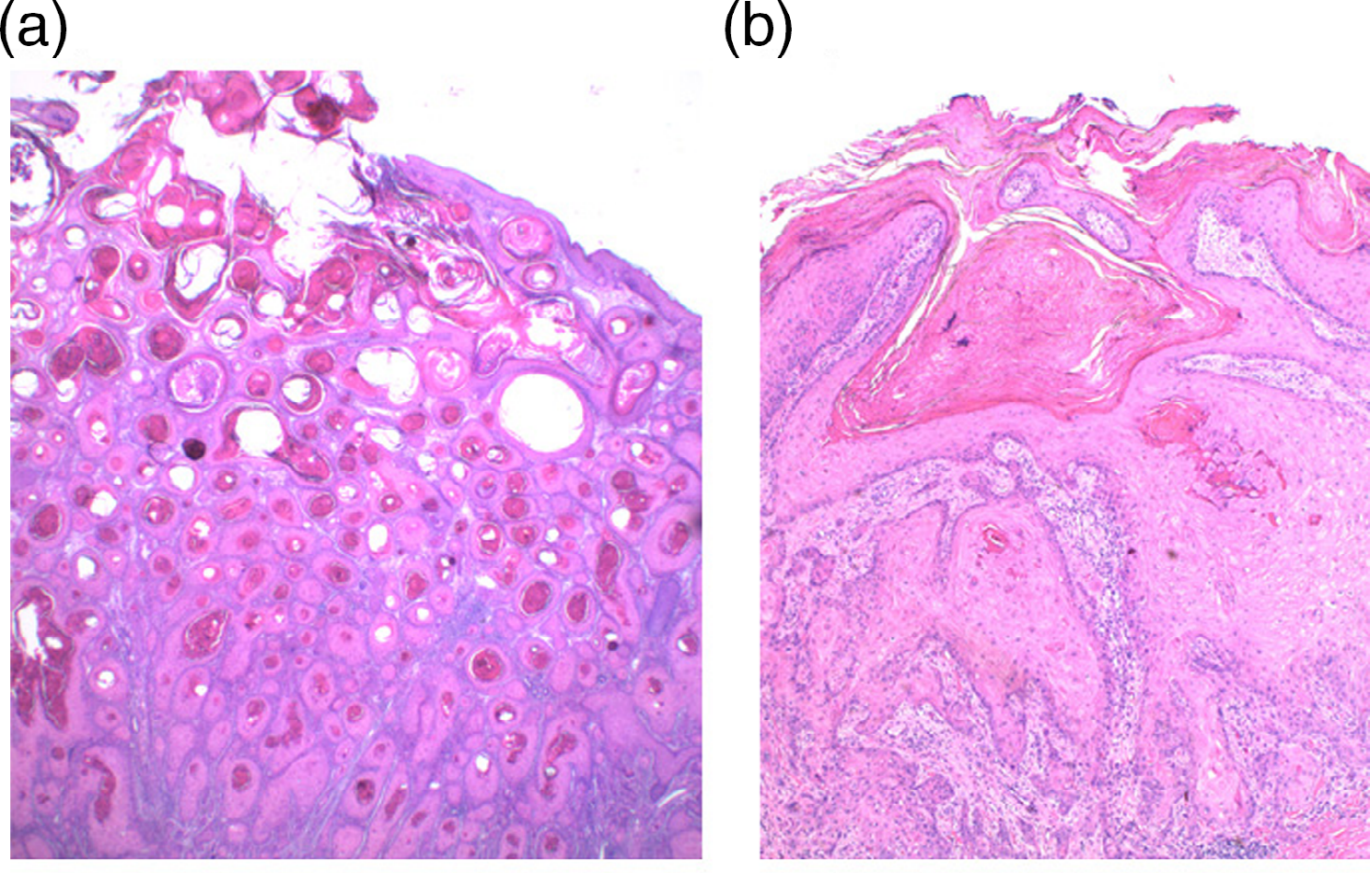
Contrast of, A, keratoacanthoma dominated by infundibulosquamous differentiation and infundibular keratinisation and, B, tumor dominated by squamoproliferation with enlarged glassy keratinocytes and trichilemmal‐like keratinisation (H&E, x50)

**FIGURE 2:**
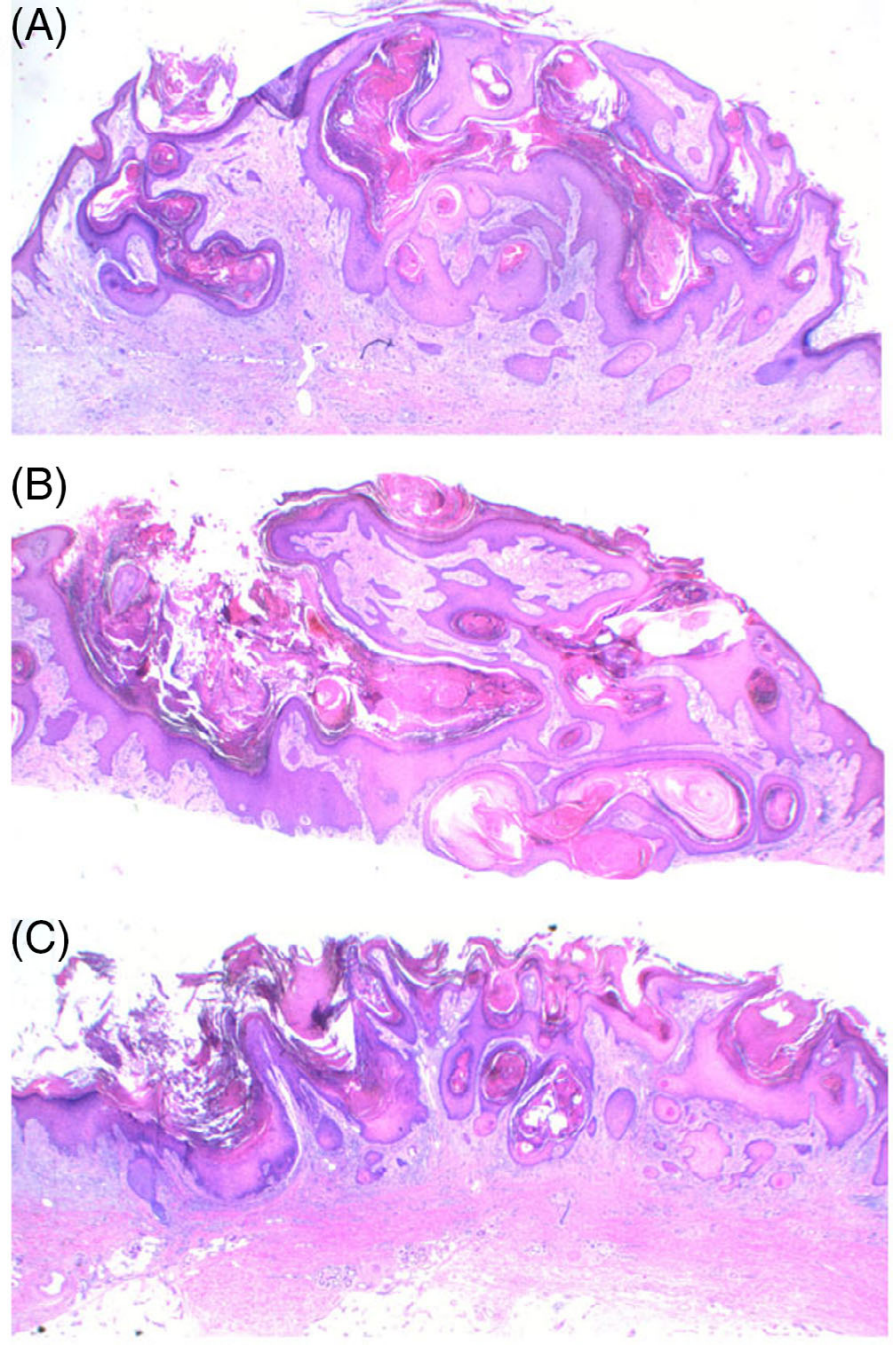
A, B, Precursors with convoluted and complex infundibulocystic hyperplasia with prominent keratinization and minimal squamoproliferation or atypia (H&E, x12.5) and, C, broad‐based precursor demonstrating prominent irregular papillomatosis with infundibulosquamous proliferation and early invasive squamous cell carcinoma (H&E, x12.5)

**FIGURE 3:**
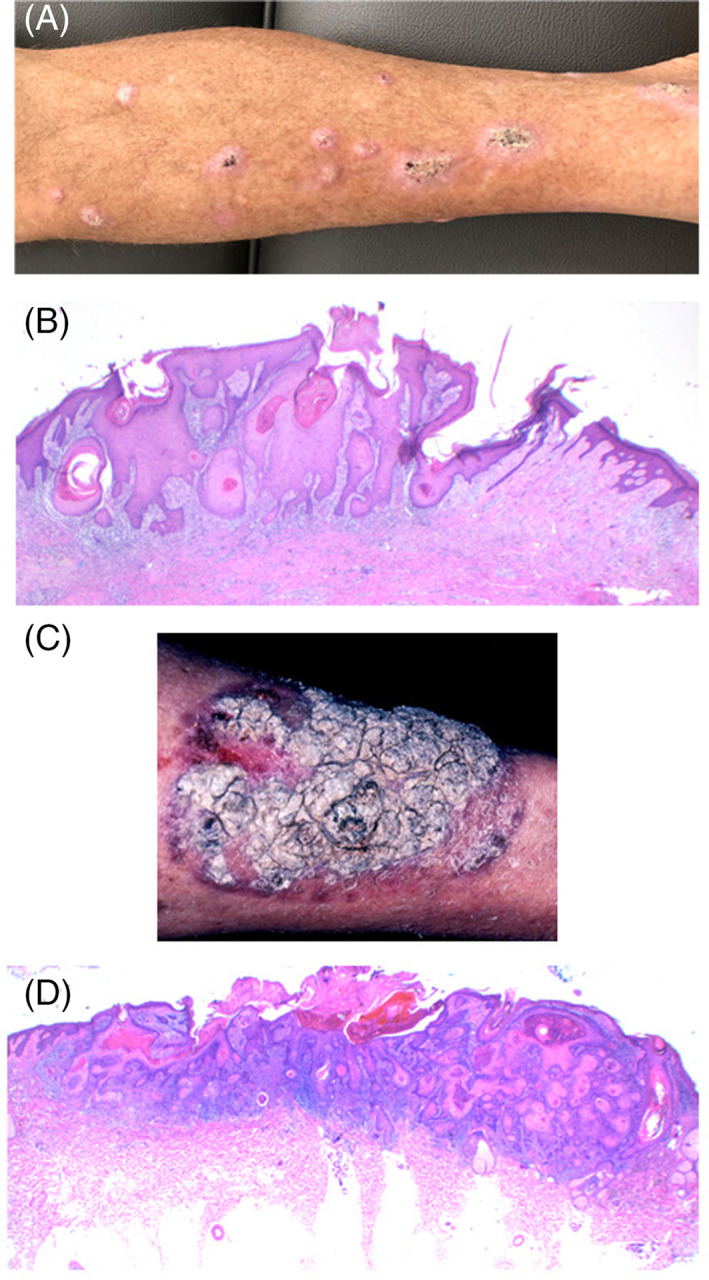
A, Multiple keratoacanthomas on lower limb. B, Biopsy of early tumor with papillomatosis, infundibulocystic squamoproliferation extending into the upper dermis over a broad front (H&E, x12.5). C, Hyperkeratotic verrucous plaque that developed within skin graft for prior keratotic squamous cell carcinoma. D, Broad‐based verrucous and infundibulocystic carcinoma (H&E x12.5)

**FIGURE 4:**
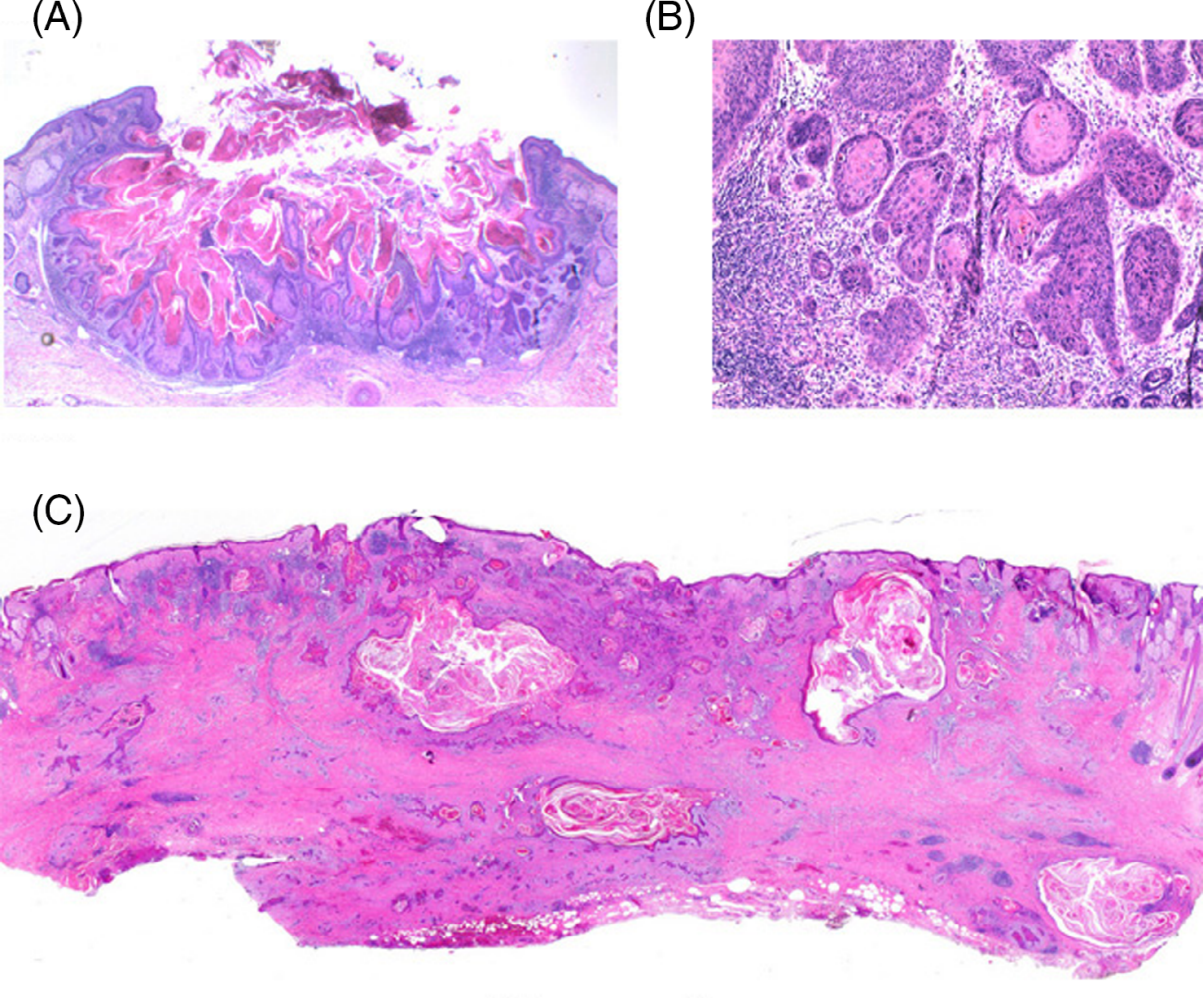
A, Crateriform nonulcerated squamous cell carcinoma with papillomatous and keratotic cavity without prominent infundibular or infundibulocystic differentiation (H&E, x12.5). B, Detail of base with invasive cords with full‐thickness atypia and minimal maturation through enlarged keratinocytes (H&E, x50). C, Broad base deeply and widely invasive well‐differentiated infundibulocystic squamous cell carcinoma with fibrosis and lichenoid inflammation (H&E, x12.5)

Infundibulocystic and infundibular SCC were diagnostic terms proposed to define a pathway to a distinct subset of SCC incorporating KA and a range of verrucous and infundibular precursors distinct from solar keratoses or Bowen disease.^1^ KAs were viewed as a form of well‐differentiated infundibulocystic SCC. Infundibulogenesis and squamoproliferation are combined in the evolution of KAs and SCCs and each can dominate (Figure 1A, B). This reflects that for committed infundibular stem cells, squamoproliferation is integral to infundibulogenesis. Complex infundibulogenesis characterizes the early phase and may have distorted infundibular hyperplasia, verrucous, and superficial squamoproliferation with lichenoid inflammation (Figure 2A‐C). The broad range of precursors is clearly apparent in eruptive squamoproliferative tumors on the lower limbs that can range from KAs to SCCs that are often not crateriform (Figure 3 A, B). Large verrucous plaques with papillomatous and infundibulocystic squamoproliferation may complicate skin grafts after KA surgery (Figure 3C, D). A subset of crateriform SCCs are likely to belong to this pathway^1^, ^12^ with the initial infundibulocystic phase rapidly subsumed by SCC producing nonulcerated craters bordered by invasive SCC (Figure 4A, B). A rarer type of SCC was also defined as a deep and broad‐based tumor retaining infundibulocystic differentiation in the deep dermis (Figure 4C).^1^

Well‐differentiated infundibulocystic SCCs including KAs dominate the pathway, but with increasing UV‐induced mutations, moderate and poorly differentiated SCCs may emerge. To be in the pathway, these tumors can only be identified by the presence of a recognizable infundibular or infundibulocystic precursors representing differentiation allied to KA. The oncogenic burden ultimately signals a departure from the committed stem cell pathway and progressive loss of recognizable committed terminal infundibular differentiation.

The increased mutational burden in the infundibulocystic pathway has been shown in the study by Ko et al.^13^ This study included 30 eruptive squamoproliferative tumors on the lower legs of 6 elderly women, 16 were classified as KA‐like squamoproliferations (KASPs), and 14 as SCCs. Utilizing *TP53* sequencing 20 of the tumors including all of the KASPs lacked mutations and 10 of 14 more cytological atypical SCCs had detectable *TP53* mutations.

## 3 KA AND ITS RELATIONSHIP TO HAIR FOLLICLES AND SCC

The link of KAs to hair follicles was demonstrated by the experimental studies by Ghadially in 1961 using topical carcinogens in mice.^4^ The early stages of these tumors emerged as infundibular‐based squamoproliferative foci within contiguous follicles that subsequently fused and produced tumors comparable to human KAs. A subset of the tumors did not involute and represented SCCs. Papillomatous and verrucous rather than crateriform tumors also emerged.

More recently, genetically engineered mouse models for human KAs have been developed.^5^ These KAs often had a component that was indistinguishable from SCC, which also involuted. Involution has been documented in humans in partially biopsied KAs showing transition to squamous cell carcinoma.^14^ This has a direct bearing on the debate of the relationship of KA to SCC as the capacity to involute or respond to retinoids is potentially shared with a much broader group of tumors within the infundibulocystic pathway.

**FIGURE 5:**
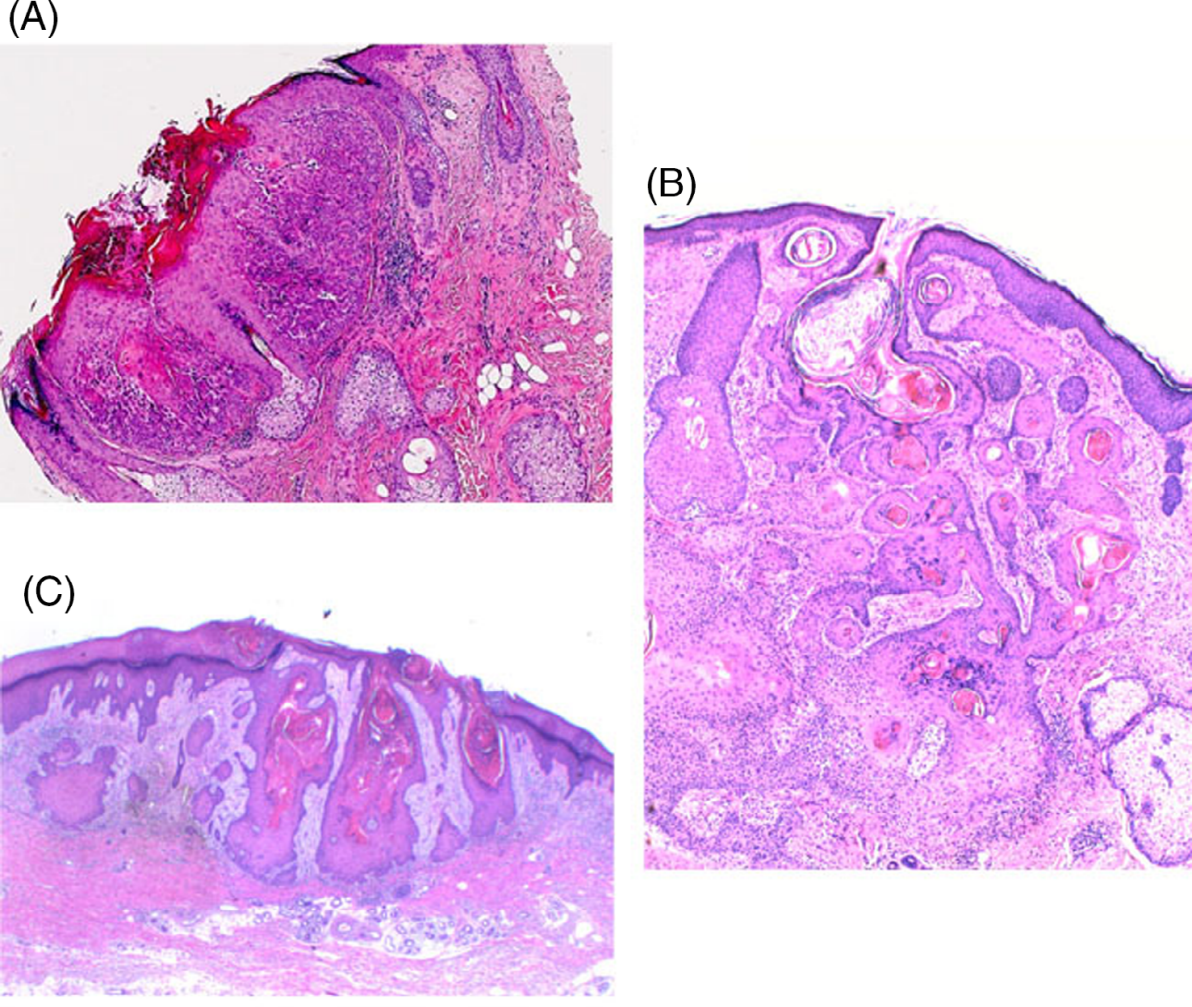
Comparison of, A, follicular squamous cell carcinoma as a nodular tumor centered on follicles with irregular solid lobules projecting into surrounding dermis (H&E, x12.5) and, B, infundibulocystic follicular squamous cell carcinoma centered on follicle with squamoproliferation and complex irregular anastomosing cords with prominent infundibular differentiation (H&E, x50)

Follicular SCCs as defined by Diaz‐Cascajo et al^15^ need to be distinguished. The SCCs in this study were not linked to KAs but as SCCs defined by their infundibular site without aberrant infundibulogenesis. These SCCs were uncommon representing only 0.14% of 7800 SCCs. The authors emphasized that they did not view these SCCs as primary follicular tumors. The importance of this study is that UV‐induced SCCs may rarely arise in the infundibulum in the absence of either solar keratoses or Bowen disease as precursors. This rare SCC variant (Figure 5A) has been subsequently broadened to include very common folliculocentric tumors dominated by infundibulogenesis and squamoproliferation allied to KA and infundibulocystic SCCs (Figure 5B, C).

The mouse model for carcinogen‐induced KAs^4^ highlighted the role of pre‐existing hair follicles. KA inception in humans may also emerge from the infundibulum of existing hair follicles, but the process is usually more complex in humans. The follicular density in human skin is a fraction seen in mouse skin and the interfollicular epidermis dominates. In humans, epidermal rather than follicular‐based infundibulogenesis with squamoproliferation is more frequent for the development of KAs and infundibulocystic SCCs.

## 4 INFUNDIBULOGENESIS AS AN EXPRESSION OF COMMITTED INFUNDIBULAR STEM CELLS

Epidermal infundibulogenesis has to be distinguished from the complex process of trichogenesis. Pluripotential stem cells within the follicular bulge and rarely from the interfollicular dermis in adults need dermal mesenchyme signaling for trichogenesis. The process is complex and is dependent on assembling a highly integrated set of differing committed stem cells that form a follicle. The identity of committed stem cells is declared through terminal differentiation. The infundibulum is one such component. *RAS*‐mutated infundibular neoplasia is not dependent on mesenchyme stroma associated with the mutated sonic hedgehog (*Shh*) pathway driving basal cell carcinomas.^9^

Misago et al^16^ extended the infundibular histogenic basis for KA to include the follicular isthmus. This conclusion was based on a study of evolving immunohistochemical keratin profiles in various stages of KA evolution excluding KA‐like SCCs. The isthmus however has no specific keratin profile and the additional keratinization pattern demonstrated in the study was akin to trichilemmal keratinization. Outer root sheath differentiation was absent in the study and both CD34 and CK19, which are expressed in normal external sheaths were negative. Keratin patterns may reflect modulation of mutated stem cells and not be a true correlate of cellular differentiation seen in normal follicles.^17^ The role of the isthmus remains important as it passages committed stem cells including those responsible for infundibular homeostasis and infundibular keratinocytes contributing to wound repair.^18^, ^19^

## 5 KA AND THE WOUND HEALING PATHWAY

Trauma has been linked to the emergence of KAs in a range of clinical scenarios.^20^, ^21^, ^22^ This event may occur in the wake of surgery including skin grafts (Figure 3A, B)^23^ radiotherapy, cryotherapy, burns and as a complication of chronic prurigo. The trauma‐prone pretibial areas are the most frequent site for UV‐induced KAs and infundibulocystic SCCs that can be eruptive. The relationship of stem cells, carcinogenesis, and the wound pathway is complex.^9^, ^20^ Particularly relevant is the Wnt/beta‐catenin pathway, which plays a critical role in skin stem cell maintenance, hair follicle development, wound healing and driving KA proliferation and subsequent terminal keratinization.^10^, ^11^

In hair‐bearing skin, wound repair is accelerated by infundibular keratinocytes driven by committed infundibular stem cells. CK15 (clones KRT15, C8/1448) was the first molecular marker for bulge‐located stem cells. This label primarily localized to the bulge has been shown to be absent in KAs^16^ and to only persist temporarily within the interfollicular epidermis following wound healing.^18^ In contrast, CK15 clone LHK 15 was shown by Misago et al^16^ to be expressed in the interfollicular epidermis and lost in KA despite being normally expressed in the follicular infundibulum and isthmus of hair follicles. Studies have found that both follicular LGR5+ cells and SOX9+ cells are also retained in the interfollicular epidermis and are activated by wounding.^9^ These retained interfollicular epidermal stem cells have the dual capacity for infundibulogenesis as well as contributing to wound repair. Conceptually, in the absence of wound healing and as a result of UV‐induced mutations or activation by biological tumor inhibitors, a subset of retained epidermal committed infundibular stem cells may undergo unscheduled keratinocyte proliferation. In the absence of a wound, the alternative committed pathway of infundibulogenesis may be engaged and depending on the mutational burden and complex driving forces, KAs, infundibulocystic SCCs and a range of other verrucous and hyperkeratotic tumors that lack or are combined with infundibulogenesis may emerge.

## 6 KA AND INVOLUTION

KAs are characterized by their capacity to involute and several potential drivers for this have been proposed. The follicular hair cycle is still considered as a driver, but it is unlikely as the infundibulum does not participate in this cycle. In addition, Wnt signaling, which regulates follicular cycling, was found to be switched off utilizing immunofluorescence and real‐time PCR approaches in both human and mouse regressing KAs.^10^

Immune‐mediated involution can be a contributory factor, as progressive growth of KAs has been observed in immunosuppressed individuals. Although lichenoid inflammation can occur in all stages of KAs, this is not a universal phenomenon. In the precursor phase, epidermal and infundibular hyperplasia with lichenoid inflammation may share features with hypertrophic lichen planus^23^ but is often verrucous with signs of infundibulogenesis and lacks clinical criteria for lichen planus. The presence of lichenoid inflammation in precursors and its persistence in the squamoproliferative stage does not always equate to regression or prevent tumor progression to SCC. A contributory role to involution is, however, supported by the presence of lichenoid inflammation when it occurs in the late involuting phase that can also target the infiltrative component signaling transition to SCC. The dynamics of KA involution are aided by the very nature of the structural progression of KAs to a crater facilitating the final elimination of the massed keratin.

The role of stem cells as an essential component in neoplasia continues to be a central focus of research.^24^ ^.^ Ultimately, the basis for involution in KA may also depend on the fate of mutated committed infundibular stem cells and their daughters in driving the infundibulocystic pathway. Committed cutaneous stem cells have a finite life cycle following an amplifying phase with rapid proliferation and differentiation, which in KAs culminates with terminal keratinization. Involution in the infundibular pathway may reflect the limited lifecycle of mutated stem cells that remain within the committed pathways. On the other hand, variants of KA such as KA centrifugum marginatum and verrucous plaque forms, appearing as multiple contiguous identical tumors, may be an expression of daughter committed stem cells. These have the capacity to migrate within the epidermis and repeat committed differentiation pathways to produce identical involuting tumors resulting in these distinct variants.

## 7 RETINOID THERAPY IN KA AND INFUNDIBULOCYSTIC SCC

Retinoids have been used to treat and slow down the appearance of multiple KAs and SCCs particularly those that are not amenable for surgery. This modality has been investigated in a pivotal study using a mouse‐based KA model.^11^ The investigators found that self‐regressing tumors shift into terminal differentiation during regression. The Wnt follicular signaling pathway sustains the growth phase of KAs and retinoids induced regression by slowing proliferation and promoting differentiation and terminal keratinization. Importantly, the retinoids were found to also induce regression in associated SCCs. This has implications for the nonsurgical treatment of the wider group of infundibular and infundibulocystic SCCs that may not evolve from KAs but arise within the wider spectrum of broadly based infundibulocystic and verrucous precursors.

## 8 ACTIVATION OF THE INFUNDIBULOCYSTIC PATHWAY TO SCC BY BRAF AND PD‐1 INHIBITORS

The introduction of BRAF and PD‐1 inhibitors for the treatment of advanced melanoma harboring V600E *BRAF* mutations has revolutionized the treatment of metastatic disease.^25^ Paradoxically one of the most frequent side effects is the emergence of cutaneous squamoproliferative lesions.^26^, ^27^, ^28^ KAs have been frequently noted and represent a surrogate marker for the infundibulocystic pathway. Keratotic papular and verrucous lesions clinically overlap with KAs.^29^ The histopathology of these tumors has been dominantly KAs, KA‐like squamoproliferative tumors or SCCs.

The emergence of KAs and SCCs as an unexpected side effect of BRAF inhibitor therapy has resulted in detailed investigative studies to discover the basis of this paradoxical induction. The studies have revealed that BRAF inhibitors result in paradoxical increased MAPK signaling and tumor development through the activation of *RAS* mutations in *BRAF* wild‐type tumor cells. A variety of oncogenic mutations including *HRAS*, *TP53*, CDKN*2A*, and *TGFBR1* have been detected within the activated *RAS‐RAF* pathway in both KA and KA‐like SCCs.^30^, ^31^ Significantly mutations could also be found in in a subset of early precursors that were infundibulocystic supporting an oncogenic pathway with increased mutational burden. In general, these papular keratotic tumors appear rapidly but are often indolent, have not been destructive or metastasized and can regress. The combining of a MEK inhibitor with the BRAF inhibitor has been shown to prevent the emergence of these tumors.^32^

Topical BRAF inhibitors in wound healing mice models have been shown to accelerate wound healing through similar paradoxical MAPK activation.^33^ This may also activate unscheduled keratinocyte proliferation in patients receiving BRAF inhibitors and in the absence of skin wounding stimulate epidermal committed infundibular stem cells to form tumors linked to the infundibulocystic pathway. In wound healing, committed infundibular stem cells are engaged in squamoproliferation and infundibulogenesis usually does not occur except in a mutated pathway or in the context of pseudo‐epitheliomatous hyperplasia. One of the main concerns at the time when *RAS*‐mutated cutaneous SCCs emerged as a consequence of BRAF inhibitor therapy was the risk of noncutaneous SCCs to emerge as up to 25% of these SCCs also harbor oncogenic *RAS* mutations. This has not eventuated and may indicate that this event is particularly restricted to the cutaneous infundibulocystic pathway. Furthermore, inherent in the infundibulocystic pathway is the capacity of KAs and the majority of the broader group of SCCs, developing as a consequence of BRAF inhibitor therapy, to involute. These tumors can be easily removed surgically or in the setting of multiple or eruptive tumors to be treated or inhibited with oral retinoids.^34^

The infundibulocystic pathway to KAs and SCCs can also be activated by programmed cell death 1 (PD‐1) inhibitors.^35^ PD‐1 inhibitors activate cytotoxic lymphocytes to target metastatic tumors including SCCs. This indicates that the role of lymphocytes in the infundibulocystic pathway is probably to be complex. Lymphocyte‐mediated lichenoid inflammation is usually viewed as a mode of tumor regression. However, the emergence of cutaneous tumors as a result of PD‐1 inhibitor therapy may reflect the capacity of lymphocytes to play an alternate role through activating proliferation of committed infundibular stem cells.

## 9 A CONCEPTUAL PATHWAY FOR NAVIGATING THE CONTROVERSIES AND PARADOXES LINKED TO KA

In essence, the conceptual infundibular and infundibulocystic pathway to SCC provides a framework to explore the controversies and paradoxes linked to KA. The term infundibulocystic was chosen on purpose to include KA as an integral member of the pathway and to highlight its infundibular and ultimately infundibulocystic structure leading to paradoxical involution. Much effort has been spent on isolating KA as a distinct entity from SCC. The introduction of a conceptual pathway means that there are no sharp boundaries and this is borne by the difficulty of setting a firm boundary between KA and SCC with the common diagnostic category KA‐like SCC. The failure of this quest supports the concept of a pathway with KA as a clinically and histopathologically recognizable member within the pathway.

A comparative study of KA and SCC utilizing comparative DNA microarray analysis revealed that KAs preferentially expressed genes that upregulate the cell death/apoptosis pathway in comparison to SCCs.^36^ This confirms that KA's have distinct features but does not indicate that they are not SCC's within the infundibulocystic pathway.

The reason for the emergence of the controversies in the relationship of KA and SCC is probably to be directly related to the complex nature of the conceptual pathway combining committed infundibular stem cells, UV‐induced *RAS*‐mutated KA and SCC and the Wnt/beta‐catenin pathway. This is not a linear pathway as the burden of UV‐ induced *RAS* mutations may at any phase overcome the involutional capacity linked to infundibulogenesis.

The focus on committed skin stem cells was initially triggered by a rare case presentation of KA centrifugum marginatum, dominated by verrucous and acanthotic gross keratoses without infundibulogenesis, limited to a finger.^37^ The complex presentation was a challenge to analyze without formulating a stem cell‐based model. The importance of this case is that the early precursor stage of the pathway that is verrucous, squamoproliferative and keratotic without infundibulogenesis was sufficient to present clinically as KA centrifugum marginatum appearing as benign nodules with gross keratinization that did not readily involute.

The separation of the infundibulocystic stem cell pathway to SCC from other committed epidermal derived stem cell pathways to SCC may not be simple and needs further exploration. It is possible that the infundibulocystic pathway may also be secondarily activated as an innate response to SCCs emerging from these alternative mutated pathways and is not always a primarily mutated infundibulocystic event.

This conceptual pathway potentially provides further areas to explore the many facets linked to the complex nature of KA particularly as a longstanding member of a broad group of tumors including SCC.

## CONFLICT OF INTEREST

The author declares no conflicts of interest.

## Data Availability

No new data was generated in this review leading to a proposed conceptual infundibulocystic pathway as this was based on published studies and the author's analysis of the controversies linked to keratoacanthoma.
